# Impact of COVID-19 Infection Rates on Pregnancy Outcomes and Disparities in Florida

**DOI:** 10.1007/s10995-025-04184-6

**Published:** 2025-10-28

**Authors:** Patrick Bernet, Sezen O. Onal

**Affiliations:** 1 Present Address: Department of Interprofessional Health Sciences and Health Administration, School of Health and Medical Sciences, Seton Hall University, 123 Metro Boulevard, Nutley, NJ 07110 USA; 2 Center for Public Health Systems, University of Minnesota, MN 55455 Minneapolis, U.S.

**Keywords:** COVID-19, Racial and ethnic disparities, Preterm birth, Low birthweight, Pregnancy

## Abstract

**Objectives:**

This study investigated whether county COVID-19 infection rates during the first trimester were associated with adverse pregnancy outcomes and whether those disproportionately impacted Black or Hispanic women.

**Methods:**

This study used birth outcomes data 2018 through 2022 for four of Florida’s five largest counties. Outcomes were paired with census tract socioeconomic characteristics and with COVID-19 infection rates during the first trimester in the woman’s home county. Outcome measures included preterm birth, low birthweight and very low birthweight. Multivariate regression was used to test the association between infection rates and all outcomes. Then, a difference-in-difference approach was used to assess the impact of infection rates on racial and ethnic outcome disparities.

**Results:**

County infection rates during the first trimester were significantly associated with worse pregnancy outcomes for all women. Each 1% point increase in COVID-19 cases during the first trimester was associated with a 5.16% point increase in the probability of preterm birth, a 4.35% point increase in the probability of low birth weight, and a 2.59% point increase in the probability of very low birth weight. Compared to White women, each 1% point increase in cases of COVID-19 during the first trimester caused a 1.21% point increase in the probability of preterm births, a 1.57% point increase in the probability of low birthweight, and a 1.28% point increase in the probability of very low birthweight among Black women. While no significant differences were observed in the probabilities of preterm birth and low birthweight between White and Hispanic women, the result revealed that each 1% point increase in cases of COVID-19 during the first trimester caused a 0.23% point increase in the probability of very low birthweight among Hispanic women compared to White women.

**Conclusions for Practice:**

This study found evidence that local COVID-19 infection rates during the first trimester are associated with worse pregnancy outcomes. Moreover, the findings indicate that local COVID-19 infection rates during the first trimester exacerbate racial disparities in these outcomes.

**Supplementary Information:**

The online version contains supplementary material available at 10.1007/s10995-025-04184-6.

## Significance

Until now, most studies of pregnancy outcomes during the COVID-19 employed a simple two-era model, with mixed findings. By linking local infection rates with pregnancy outcomes, this study provides conclusive evidence that there is a significant dose-and-response style association with worse pregnancy outcomes and heighted disparities among Black women. These findings inform policy decisions regarding public health measures to inhibit COVID-19 infection surges. Looser safeguards will be associated with worse pregnancy outcomes, and the high costs associated with adverse outcomes will healthcare spending; much of which is publicly funded.

**Supplementary Information:**

The online version contains supplementary material available at 10.1007/s10995-025-04184-6.

## Introduction

Prenatal stressful life events have been associated with worse pregnancy outcomes by many studies (Kim, 2022). A meta-analysis of 31 studies involving 5,665,998 pregnant women found a 20% increase in preterm births (PTB) and a 23% increase in low birthweight (LBW; birthweight < 2500 g) outcomes among women exposed to stressful life events (Ding et al., 2021). Multiple studies show that pandemic was associated with significant increases in levels of distress, anxiety and depression(Manchia et al., 2022).

There is mixed evidence on the association of being pregnant during the pandemic and birth outcomes. Compared to pregnancies prior to the pandemic, many large studies find outcomes were better during the pandemic (Caniglia et al., 2021; Yao et al., 2022). One study found the odds of extreme preterm birth (PTB; <37 weeks gestation) were significantly lower, citing decreased physical stress during lockdown periods as a potential contributor (Morgan et al., 2022). Another study of 1.60 million births found a significant drop in PTB in the 4 months following the start of the pandemic (Been et al., 2020). A meta-analysis of 63 separate studies found the odds of PTB dropped significantly between prepandemic and pandemic births; even when the women were not COVID-19 infected(Yao et al., 2022). Some studies, however, find no significant change in outcomes (Grobman et al., 2023). A large national study of 1.65 million births found no difference in PTB and infant mortality between the prepandemic (through February 2020) and pandemic eras (beginning March 2020) (Molina et al., 2022). Similarly, a large California study found PTB rates dropped substantially in the first 4 months of the pandemic (Torche & Nobles, 2022), only to rise again by early 2021.

Looking beyond these population wide PTB findings, a focus on race and ethnicity reveals disparities in work situations and health outcomes. Black and Hispanic workers were more likely employed in industries and positions that were considered essential and less likely to be able to work from home (Kearney et al., 2020). Essential jobs require in-person attendance and close interpersonal contact that increases the chance of infection. Positions include personal care, healthcare support, healthcare practitioner, cleaning and transportation (Asfaw, 2022; Faberman & Hartley, 2022; Rogers et al., 2020). Highlighting the role of workplace risks, early pandemic studies found that significant portions of COVID-19 mortality disparities between Black, Hispanics and Whites were associated with differences in employment mix (Rogers et al., 2020). Even among people in similar occupations, Black and Hispanic workers felt more fearful (Vargas et al., 2023) and were significantly more concerned about COVID-19 exposure at work (72% and 65%, respectively; compared to 43% among White workers) (Parker et al., 2022).

Despite these racial and ethnic differences in work-related stress and mortality, empirical research to-date finds no significant disparities in pregnancy outcomes during the pandemic (Janevic et al., 2021; Molina et al., 2022). Even studies that look into multiple risk factors still conclude that the lack of association is universal; the race, ethnicity, insurance status and even the community vulnerability measures were not associated with differing PTB rate changes (Grobman et al., 2023). Another study reported disproportional impact among women with lower educational attainment (Torche & Nobles, 2022), but the effect was not moderated by patient race or ethnicity. Another study went so far as to include neighborhood structural racism measures, but still found no evidence of interaction effects between COVID-19 infection and adverse pregnancy outcomes (Janevic et al., 2022).

Most studies of pregnancy outcomes during the pandemic measure effects in binary terms and don’t consider dose-response reactions based on infection levels. One study considered the gestational age at which the pandemic started (Margerison et al., 2023), finding reduced PTB risks among pregnancies that were already close-to-term when the pandemic began. Like most studies discussed here, this study acknowledges that findings contrast with the dominant narrative that pandemic-related stress was expected to increase adverse pregnancy outcomes.

Although we were unable to find large studies that match gestation age with local infection rates, prior research suggests that there may be a relationship. A systematic review identified studies in which higher rates of preterm birth were associated with stressful events during the first and second trimester, such as “a death in the family, becoming unemployed [and] natural disasters” (Shapiro et al., 2013). Using this metric, the pandemic could be considered multiple events because of increasing deaths and higher unemployment rates. Third trimester events were generally found to have no significant impact on pregnancy outcomes; generally attributed to dampened stress responses as the HPA axis downregulates over the course of gestation.

The pandemic was related to multiple potential sources of stress. In addition to worries about the illness itself, many people lost jobs, saw family members fall ill, and had to rearrange living space to create space for home offices. Prior research finds there can be a cumulative impact from multiple stresses, and that the impact is strongest during the first and second trimesters (Bond et al., 2019; Zhu et al., 2010). Another study found PTB odds were 1.42 higher if the woman changed residence in the first trimester (Bond et al., 2019). One study finds that exposure to just one stressful event (such as a sick spouse or lost job) was not significant, but exposure to two or more was associated PTB odds 2.40 times higher if exposed in the first trimester (Zhu et al., 2010).

In contrast to the timing of stress-related outcomes, many studies of individual infection find that a positive COVID-19 diagnosis during the third trimester was associated with higher rates of PTB, LBW, maternal ICU admission, maternal mortality (Doyle et al., 2022; Sevoyan et al., 2025; Son et al., 2021; Villar et al., 2021), and lower APGAR scores (Xu et al., 2024) than when the infection occurred in earlier trimesters. Paradoxically, this implies that pregnancy outcomes were less sensitive to first trimester individual infections, yet more sensitive to other psychological stressors. Much research into the impact of the pandemic on pregnancy outcomes has been done to-date, but most test pandemic effects in simple prepandemic and pandemic era terms. Rather than use such a coarse binary indicator, our study employs a continuous measure of local infection rates. If individuals perceive higher stress in response to higher infection rates, those stresses may manifest in pregnancy outcomes in much the same way as other continuous risk factors, such as education, age, BMI, and income. To advance understanding of the impact of the pandemic on pregnancy outcomes, our study tests two hypotheses. First, is the local infection rate during the first trimester related to pregnancy outcomes? Although most pandemic studies find no change in racial and ethnic disparities, our second hypothesis retests these disparities by examining whether the effects of COVID-19 infection rates are differentially experienced across racial groups by using difference-in-difference methods.

## Methods

### Data Sources

Our study uses data on 291,145 Florida births in Broward, Hillsborough, Orange, and Palm Beach counties. All singleton births from 2018 to 2022 are included. Key fields are extracted from birth certificates and summarized on the Florida HealthCHARTS data repository (Florida Department of Health (FDOH), 2020). Birth data is paired with socioeconomic characteristics (US Census Bureau, n.d.), as well as COVID-19 infection rates in the woman’s county during the first trimester (CDC, 2020). Births with missing or invalid information are excluded from analysis. The study was found to be exempt from IRB approval by Florida Atlantic University IRB because it was considered research not involving human subjects due to the use of retrospective publicly available data.

The dependent variables are individual-level PTB, LBW and very low birthweight (VLBW; <1500 g) outcomes. Many studies of pregnancy outcomes focus on these outcomes (Burns et al., 2020; Haynes & Ogneva-Himmelberger, 2020; Khan et al., 2021; Meng et al., 2013; Molina et al., 2022; Torche & Nobles, 2022). Independent variables include birth-specific characteristics that prior studies have identified as significantly associated with LBW and PTB (Amjad et al., 2019; Hibbs et al., 2018; Molina et al., 2022; Ngui et al., 2015; Torche & Nobles, 2022). Maternal age is measured in 5-year intervals; except for those under 20 and over 39. Race and ethnicity are coded as White (non-Hispanic), Black (non-Hispanic), Hispanic (of any race) and other. Education is measured simply as whether the woman graduated high school. Insurance coverage is coded as Medicaid, private health insurance or self-pay. In addition to marital status, another variable indicates if the father’s name was not listed on the birth certificate. Pre-conception body mass index (BMI) is coded as underweight (under 18.5), normal (18.5 to 24.9), overweight (25 to 29.9), and obese (30 or over).

Community level independent variables are linked via maternal census tract and include neighborhood characteristics found to be associated with pregnancy outcomes in prior studies (Been et al., 2020; Burns et al., 2020; Haynes & Ogneva-Himmelberger, 2020; Meng et al., 2013; Wang et al., 2021). Local unemployment rate is measured as the % of people aged 16 and over who are in the labor force (employed or actively seeking work), but not working. Median income is measured at the household level. Neighborhood racial and ethnic composition is measured as the percent White non-Hispanic, Black non-Hispanic, and percent neither Black nor White.

More than simply indicating pre-pandemic or pandemic era as most studies do (Molina et al., 2022; Prasannan et al., 2021), our study allows for the possibility that stress is higher during times when infection rates are higher. Further, we focus on infection rates during the first trimester because prior research identifies that as the period most sensitive to stressful life events (Bond et al., 2019; Shapiro et al., 2013; Zhu et al., 2010). Infection rate is measured as the monthly average number of infections per 100 people in the woman’s county during the first trimester (gestation weeks 1–12).

### Statistical Analysis

To assess the impact of the COVID-19 pandemic on pregnancy outcomes across different racial groups, we employed a comprehensive analytical strategy involving linear probability multivariate regression models followed by a difference-in-difference (DID) approach (Angrist & Pischke, 2009; Imbens & Wooldridge, 2009). This analysis leverages a continuous treatment variable, representing the monthly average number of local COVID-19 cases per 100 people during the first trimester within the woman’s county, to measure the pandemic’s effect. Initially, we applied linear probability models to evaluate the overall influence of COVID-19 on pregnancy outcomes without distinguishing between race or ethnic groups. This approach set a baseline understanding of the pandemic’s impacts, using the continuous variable to quantify exposure levels across the entire sample.

Building on this foundation, we introduced a DID methodology to specifically investigate racial disparities. Such methods are common when examining how changes experienced by an entire population impact child health (Janevic et al., 2018; Li et al., 2019). In this more focused analysis, the continuous treatment variable was enhanced with an interaction term between infection rates and racial / ethnic group identifiers. This crucial addition allows for a detailed examination of how the pandemic’s first trimester exposure differentially affects outcomes across White, Black, and Hispanic groups, with Whites used as the reference category. The DID design is particularly advantageous in this context as it mitigates biases from time-invariant confounding variables within racial groups and across counties. This methodological choice strengthens causal inference by focusing on internal changes within groups over time, rather than cross-sectional comparisons that could be skewed by unobserved heterogeneity.

To ensure the validity of our DID approach, we tested the parallel trends assumption, confirming that the trends in pregnancy outcomes were similar across groups prior to the pandemic. Details on the methodology and results of this test are provided in Appendix 1.

Our econometric model, employed within the framework of a DID methodology is specified as follows:

$$\begin{aligned} Y_{{ict}} \, = \, & \beta _{0} + ~\beta _{1} ~\left( {X_{{ict}} *Race_{i} } \right) + ~\beta _{2} ~X_{{ict}} + \beta _{3} Race_{i} \\ & \, + \beta _{4} Z_{{ct}} + \beta _{5} ~L_{{it}} + \gamma _{c} + \alpha _{t} + \varepsilon _{{ict}} \\ \end{aligned}$$

In this equation, the subscripts indicate individual $\:i$, $\:c$ county, and year $\:t$.

$\:{Y}_{ict}\:$represents the pregnancy outcome for woman in county *c* during year , including PTB, LBW and VLBW—each treated as a binary variable. $\:{X}_{ict}\:$represents the monthly average number of localized COVID-19 cases per 100 people during the first trimester, and $\:Rac{e}_{i}\:$is a categorical indicator of the woman’s race or ethnicity. $\:{Z}_{ct}$ is a vector of county-level controls including percentage of white, Black, and neither white nor Black population, unemployment rate, medium household income. $\:{L}_{it}$ is a vector of individual controls including woman’s educational attainment, pre-pregnancy BMI, marital status, and insurance status. All specifications also include county $\:{\gamma\:}_{c}$ and time $\:{\alpha\:}_{t}$ fixed effects.

Considering the potential for small-sample bias in standard error calculation—a known challenge in studies with limited clusters—we apply block-bootstrapped standard errors based on 200 replications (Cameron et al., 2008; Donald & Lang, 2007; McCaffrey & Bell, 2006). Given our model’s reliance on just four county clusters, this technique is crucial for obtaining reliable, robust standard errors, thus enhancing the validity of our conclusions.

## Results

Table 1 summarizes pregnancy outcomes, maternal and community characteristics during the prepandemic and pandemic eras. Although PTB rates did not change significantly among White and Hispanic women, there was a significant increase from 12.5% to 13.3% among Black women. LBW rates also increased among Black women (12.0% to 12.8%). Looking at all races and ethnicities combined, there was no significant change in PTB or VLBW.

**Table 1:** Descriptive characteristics of pregnant women and their communities in the pre-pandemic and pandemic eras

	All births			White Woman Only			Black Women Only			Hispanic Women Only		
Variable	Pre	Post		Pre	Post		Pre	Post		Pre	Post	
n	145,651	145,494		45,195	43,626		29,356	28,874		60,607	62,627	
	%	%		%	%		%	%		%	%	
Preterm (< 37 Gestation Weeks)	0.0877 (0.28)	0.0891 (0.28)		0.0672 (0.25)	0.0667 (0.25)		0.1251 (0.33)	0.1324 (0.34)	**	0.0875 (0.28)	0.0866 (0.28)	
Infant Weight at Delivery < 2500	0.0735 (0.26)	0.0761 (0.27)	**	0.0489 (0.22)	0.0505 (0.22)		0.1194 (0.32)	0.1280 (0.33)	**	0.0695 (0.25)	0.0694 (0.25)	
Infant Weight at Delivery < 1500	0.0139 (0.12)	0.0134 (0.11)		0.0064 (0.08)	0.0063 (0.08)		0.0258 (0.16)	0.0277 (0.16)		0.0142 (0.12)	0.0120 (0.11)	**
Age 18–20	0.0505 (0.22)	0.0461 (0.21)	***	0.0256 (0.16)	0.0203 (0.14)	***	0.0833 (0.28)	0.0751 (0.26)	***	0.0584 (0.23)	0.0555 (0.23)	
Age 21–24	0.1340 (0.34)	0.1258 (0.33)	***	0.0889 (0.28)	0.0771 (0.27)	***	0.1910 (0.39)	0.1802 (0.38)	**	0.1492 (0.36)	0.1439 (0.35)	**
Age 25–29	0.2733 (0.45)	0.2595 (0.44)	***	0.2556 (0.44)	0.2350 (0.42)	***	0.3002 (0.46)	0.2912 (0.45)		0.2758 (0.45)	0.2659 (0.44)	***
Age 30–34	0.3064 (0.46)	0.3209 (0.47)	***	0.3695 (0.48)	0.3869 (0.49)	***	0.2383 (0.43)	0.2560 (0.44)	***	0.2820 (0.45)	0.2954 (0.46)	***
Age 35–39	0.1789 (0.38)	0.1902 (0.39)	***	0.2071 (0.41)	0.2281 (0.42)	***	0.1324 (0.34)	0.1446 (0.35)	***	0.1735 (0.38)	0.1770 (0.38)	
Age 40+	0.0475 (0.21)	0.0491 (0.22)		0.0495 (0.22)	0.0500 (0.22)		0.0381 (0.19)	0.0383 (0.19)		0.0496 (0.22)	0.0517 (0.22)	
Race/Ethnicity White Non-Hispanic	0.3103 (0.46)	0.2998 (0.46)	***	1.0000 (0.00)	1.0000 (0.00)							
Race/Ethnicity Black Non-Hispanic	0.2016 (0.40)	0.1985 (0.40)					1.0000 (0.00)	1.0000 (0.00)				
Race/Ethnicity Other Non-Hispanic	0.0720 (0.26)	0.0713 (0.26)										
Race/Ethnicity Hispanic (Any Race)	0.4161 (0.49)	0.4304 (0.50)	***							1.0000 (0.00)	1.0000 (0.00)	
Education Attainment Below HS	0.1611 (0.37)	0.1499 (0.36)	***	0.0507 (0.22)	0.0426 (0.20)	***	0.1803 (0.38)	0.1537 (0.36)	***	0.2704 (0.44)	0.2587 (0.44)	***
Education Attainment HS	0.2389 (0.43)	0.2269 (0.42)	***	0.1911 (0.39)	0.1675 (0.37)	***	0.3493 (0.48)	0.3448 (0.48)		0.2501 (0.43)	0.2441 (0.43)	
Education Attainment above-HS	0.6000 (0.49)	0.6232 (0.48)	***	0.7582 (0.43)	0.7899 (0.41)	***	0.4703 (0.50)	0.5015 (0.50)	***	0.4796 (0.50)	0.4972 (0.50)	***
Education Attainment Associates	0.1397 (0.35)	0.1393 (0.35)		0.1253 (0.33)	0.1188 (0.32)	**	0.1689 (0.37)	0.1696 (0.38)		0.1459 (0.35)	0.1482 (0.36)	
Education Attainment Bachelor	0.2994 (0.46)	0.3099 (0.46)	***	0.4004 (0.49)	0.4168 (0.49)	***	0.1978 (0.40)	0.2182 (0.41)	***	0.2345 (0.42)	0.2405 (0.43)	
Education Attainment Master +	0.1609 (0.37)	0.1740 (0.38)	***	0.2325 (0.42)	0.2543 (0.44)	***	0.1037 (0.30)	0.1137 (0.32)	**	0.0992 (0.30)	0.1085 (0.31)	***
BMI Pre-Pregnancy Underweight	0.0358 (0.19)	0.0304 (0.17)	***	0.0386 (0.19)	0.0346 (0.18)	**	0.0365 (0.19)	0.0302 (0.17)	***	0.0294 (0.17)	0.0248 (0.16)	***
BMI Pre-Pregnancy Normal	0.4329 (0.50)	0.4114 (0.49)	***	0.5171 (0.50)	0.5039 (0.50)	***	0.3404 (0.47)	0.3191 (0.47)	***	0.3969 (0.49)	0.3720 (0.48)	***
BMI Pre-Pregnancy Overweight	0.2814 (0.45)	0.2862 (0.45)	**	0.2478 (0.43)	0.2529 (0.43)		0.2710 (0.44)	0.2729 (0.45)		0.3148 (0.46)	0.3176 (0.47)	
BMI Pre-Pregnancy Obese	0.2474 (0.43)	0.2694 (0.44)	***	0.1932 (0.39)	0.2049 (0.40)	***	0.3496 (0.48)	0.3755 (0.48)	***	0.2571 (0.44)	0.2839 (0.45)	***
Married?	0.5658 (0.50)	0.5527 (0.50)	***	0.7231 (0.45)	0.7363 (0.44)	***	0.3111 (0.46)	0.2918 (0.45)	***	0.5317 (0.50)	0.5058 (0.50)	***
Father Name Missing	0.0899 (0.29)	0.1022 (0.30)	***	0.0414 (0.20)	0.0466 (0.21)	***	0.2066 (0.40)	0.2383 (0.43)	***	0.0784 (0.27)	0.0881 (0.28)	***
Payor - Medicaid	0.4488 (0.50)	0.4331 (0.50)	***	0.2644 (0.44)	0.2300 (0.42)	***	0.6537 (0.48)	0.6279 (0.48)	***	0.5183 (0.50)	0.5146 (0.50)	
Payor - Private	0.4441 (0.50)	0.4861 (0.50)	***	0.6460 (0.48)	0.7012 (0.46)	***	0.2887 (0.45)	0.3269 (0.47)	***	0.3399 (0.47)	0.3800 (0.49)	***
Payor - Self Pay	0.0874 (0.28)	0.0646 (0.25)	***	0.0650 (0.25)	0.0504 (0.22)	***	0.0437 (0.20)	0.0336 (0.18)	***	0.1230 (0.33)	0.0893 (0.29)	***
% Not Hispanic (White race only)	38.1 (22.57)	38.9 (22.55)	***	52.6 (20.46)	53.5 (20.37)	***	24.9 (19.29)	26.4 (19.47)	***	33.0 (19.81)	33.7 (19.93)	***
% Not Hispanic (Black race only)	24.9 (23.98)	24.1 (23.42)	***	13.8 (14.71)	13.3 (14.37)	***	44.7 (28.16)	42.2 (27.93)	***	24.6 (22.14)	24.4 (21.96)	
% Not White nor Black	6.9 (5.14)	6.9 (5.12)	**	7.3 (5.27)	7.4 (5.22)		5.9 (4.79)	6.1 (4.84)	***	6.5 (4.79)	6.6 (4.83)	
% Unemployment	5.6 (3.98)	5.5 (3.92)	***	4.6 (3.34)	4.6 (3.32)	**	7.2 (4.73)	6.9 (4.61)	***	5.7 (3.86)	5.6 (3.82)	**
Median HH Income Total	$63,957 (27762)	$64,896 (28197)	***	$75,695 (30078)	$77,224 (30823)	***	$51,924 (21543)	$53,284 (21920)	***	$59,587 (24505)	$60,337 (24909)	***
COVID infect rate in Pregnancy trimester 1	0.00 (0.00)	0.6235 (0.0015)	***	0.00 (0.00)	0.6130 (0.0025)	***	0.00 (0.00)	0.6320 (0.0035)	***	0.00 (0.00)	0.6285 (0.0020)	***

Time periods: “Pre” includes Jan-2018 to Feb-2020; “Post” includes Mar-2020 to Dec-2022

Includes all singleton births in Florida counties: Broward, Hillsborough, Orange, and Palm Beach

*,**,***: t-test is significantly different from zero at the 0.05, 0.01, 0.001 level, respectively

Each cell contains variable mean, with standard deviations in parentheses

Average maternal age increased in the pandemic era. Women below 30 years old comprised 45.9% of all singleton births prepandemic and 43.2% in the pandemic era. The proportionate shift was strongest among White women (37% to 33%), though Black women were still younger on average in both periods. The racial and ethnic composition of births changed also, with Hispanic women increasing their relative share (41.6% to 43.0%) as White and Black shares decreased slightly.

Average education levels also increased in the pandemic era. Overall, the proportion of births to women with more than a high school level of education increased from 60.0% to 62.3%. The proportion of births to women with higher preconception BMIs increased during the pandemic, with the obese BMI range increasing the most. The share of births to White women with obese preconception BMIs increased from 19.3% to 20.5%; Among Hispanic women the share rose from 25.7% to 28.4%; And among Black women, the share expands from 35.0% to 37.6%.

There was an increase in births to married White women and a decrease among Black and Hispanic women. The share of births for which the father was not listed on the birth certificate increased during the pandemic (9% to 10.2% overall), with the largest increase among Black women (20.7% to 23.9%). The payor share of births shifted dramatically during the pandemic, with Medicaid’s share decreasing (44.9% to 43.3%) and private insurer share increasing (44.4% to 48.6%). Turning to community characteristics, the share of births shifted towards women living in census tracts that had higher proportions of White women (38.1% to 38.9%), with lower unemployment (5.6 to 5.5%), and higher median household income ($63,956 to $64,896).

Table 2 displays the results of linear probability multivariate regressions for PTB, LBW and VLBW among women of all races and ethnicities. There was a strong association between county infection rates during the first trimester and adverse birth outcomes. Each 1% point increase in COVID-19 cases was associated with percentage point increases of 5.16 in PTB, 4.35 in LBW, and 2.59 In VLBW (*P* < 0.001). Turning to maternal characteristics, PTB probabilities were 3.38% points higher among Black women (*p* < 0.001) and 0.72% points higher for Hispanic women (*p* < 0.01) relative to White non-Hispanic women. LBW probabilities were 4.51% points higher for Black women (*p* < 0.001) and 0.62% points higher for Hispanic women (*p* < 0.05). Women above the age of 34 had higher odds of PTB, LBW and VLBW relative to the 30–34 reference age interval. Women over 39 years old had the highest risks, with PTB probabilities 4.26% points higher; 3.18% points higher for LBW and 0.71% points higher for VLBW (*p* < 0.001). Relative to women with education levels above high school levels, those with HS-level education had a 1.09% points increased probability of PTB (*p* < 0.001), 0.57% points higher for LBW (*p* < 0.05) and 0.012% points higher for VLBW (*p* < 0.05). High pre-pregnancy BMIs were associated with higher odds of PTB and VLBW. Compared to normal BMI women, obese women had 2.26% points higher probabilities of PTB, and a 0.41% points higher risk of VLBW.

**Table 2:** Pregnancy outcomes, maternal characteristics, and COVID-19 infection rates (2018 to 2022)

All births	PTB		LBW		VLBW	
COVID-19, Race, Ethnicity						
COVID infect rate in Pregnancy trimester 01	0.0516	***	0.0435	***	0.0259	***
	(0.0041)		(0.0046)		(0.0035)	
Race/Ethnicity Black Non-Hispanic	0.0338	***	0.0451	***	0.0129	***
	(0.0017)		(0.0011)		(0.0004)	
Race/Ethnicity Hispanic (Any Race)	0.0072	**	0.0062	*	0.0032	***
	(0.0024)		(0.0027)		(0.0005)	
Maternal						
Age 00–19	− 0.0107	*	0.0000		− 0.0013	
	(0.0047)		(0.0058)		(0.0023)	
Age 20–24	− 0.0167	***	− 0.0069	**	− 0.0033	***
	(0.0032)		(0.0025)		(0.0006)	
Age 25–29	− 0.0095	***	− 0.0050	***	− 0.0017	***
	(0.0006)		(0.0011)		(0.0005)	
Age 35–39	0.0152	***	0.0091	***	0.0023	***
	(0.0029)		(0.0014)		(0.0006)	
Age 40-up	0.0426	***	0.0318	***	0.0071	***
	(0.0019)		(0.0021)		(0.0017)	
Education Attainment sub-HS	0.0146	***	0.0094		− 0.0005	
	(0.0042)		(0.0052)		(0.0012)	
Education Attainment HS	0.0109	***	0.0057	*	0.0012	*
	(0.0030)		(0.0028)		(0.0005)	
Pre-Pregnancy BMI Underweight	0.0209	***	0.0354	***	0.0041	***
	(0.0032)		(0.0054)		(0.0005)	
Pre-Pregnancy BMI Overweight	0.0039	***	− 0.0057	***	0.0012	**
	(0.0007)		(0.0005)		(0.0004)	
Pre-Pregnancy BMI Obese	0.0226	***	− 0.0013		0.0041	***
	(0.0038)		(0.0016)		(0.0004)	
Insurance and Family						
Payor - Medicaid	0.0043	**	0.0058	**	0.0009	
	(0.0013)		(0.0019)		(0.0027)	
Payor - Private	− 0.0042		− 0.0006		− 0.0017	
	(0.0035)		(0.0027)		(0.0028)	
Payor - Self Pay	− 0.0242	***	− 0.0179	***	− 0.0060	*
	(0.0046)		(0.0031)		(0.0028)	
Married	− 0.0052	***	− 0.0083	***	− 0.0010	
	(0.0014)		(0.0013)		(0.0007)	
Father Name Missing	0.0284	***	0.0228	***	0.0053	***
	(0.0012)		(0.0023)		(0.0010)	
Community						
% Not Hispanic (White race only)	− 0.00009	*	− 0.00003		− 0.00002	*
	(0.00004)		(0.00003)		(0.00001)	
% Not Hispanic (Black race only)	0.00021	***	0.00030	***	0.00006	***
	(0.00005)		(0.00004)		(0.00002)	
% Not White nor Black	− 0.00010		0.00011	**	− 0.00004	
	(0.00021)		(0.00004)		(0.00003)	
% Unemployment	0.0002		0.0004	**	0.0000	
	(0.0002)		(0.0001)		(0.0001)	
Median household income (ln)	− 0.0047	**	− 0.0053	*	− 0.0019	***
	(0.0016)		(0.0025)		(0.0003)	
Controls						
Infant DOB Year = 2019	0.0034	***	− 0.0008		0.0000	
	(0.0007)		(0.0005)		(0.0006)	
Infant DOB Year = 2020	− 0.0008		− 0.0026		− 0.0020	**
	(0.0009)		(0.0014)		(0.0008)	
Infant DOB Year = 2021	− 0.0377	***	− 0.0340	***	− 0.0221	***
	(0.0077)		(0.0070)		(0.0044)	
Infant DOB Year = 2022	− 0.0585	***	− 0.0484	***	− 0.0315	***
	(0.0066)		(0.0051)		(0.0040)	
Constant	0.1120	***	0.1070	***	0.0282	***
	(0.0161)		(0.0265)		(0.0038)	
N	194,123		194,123		194,123	
aic	44,102		11,892		− 317,000	
bic	44,387		12,177		− 316,000	

Time periods: “Pre” includes Jan-2018 to Feb-2020; “Post” includes Mar-2020 to Dec-2022. Includes all singleton births in Florida counties: Broward, Hillsborough, Orange, and Palm Beach. Jan-2018 to Dec-2022

Base levels: Infant DOB (date of birth) base year is 2018. Maternal age base is 30–34. Pre-Pregnancy BMI base level is “Normal”. Education attainment base level is “over high school” (associates, bachelors, etc.)

*,**,***: t-test is significantly different from zero at the 0.05, 0.01, 0.001 level, respectively

Standard deviations in parentheses

Medicaid coverage was associated with slightly higher risks of PTB and LBW (0.43 and 0.58% points, respectively), while self-pay was associated with much lower probabilities of PTB (-2.42% points *p* < 0.001), LBW (-1.79% points, *p* < 0.001), and VLBW (-0.60% points, *p* < 0.05). Marriage was associated with lower PTB and LBW probabilities (respectively − 0.52 and − 0.83% points, *p* < 0.001). Births for which the father’s name was missing from the birth certificate are associated with significant increases in the probabilities of PTB (2.84% points), LBW (2.28% points), and VLBW (0.53% points, *p* < 0.001).

The community characteristic most strongly associated with outcomes was the racial mix, with each additional percent in Black population associated with percentage point increases of 0.021 in PTB, 0.030 in LBW, and 0.006 in VLBW (*p* < 0.001). Higher household incomes were associated with decreased probabilities of PTB, LBW and VLBW.

Table 3 displays the results of the DID analyses, focusing just on infection rates, race and ethnicity. The first set of models compares Black and White women. For births before and during the pandemic, Black women had higher rates of PTB (3.14% points), LBW (4.48% points), and VLBW (1.03% points, *p* < 0.001). For births to both Black and White women, each 1% point increase in Covid-19 cases during the first trimester were associated with percentage point increases of 5.25 in PTB, 4.24 in LBW, and 2.35 in VLBW (*p* < 0.001). The interaction term shows same increment in cases was associated with an additional percentage point increase in PTB (1.21), LBW (1.57), and VLBW (1.28, *p* < 0.001) among Black women.

**Table 3:** Pregnancy outcomes, COVID-19 infection rates and racial/ethnic interaction effects (2018 to 2022)

a. Births to White and Black women	PTB		LBW		VLBW	
COVID-19, Race, Ethnicity						
COVID infect rate in Pregnancy trimester 01	0.0525	***	0.0414	***	0.0235	***
	(0.0043)		(0.0044)		(0.0045)	
Race/Ethnicity Black Non-Hispanic	0.0314	***	0.0448	***	0.0103	***
	(0.0028)		(0.0029)		(0.0006)	
COVID infect rate trimester 01 x Black	0.0121	***	0.0157	***	0.0128	***
	(0.0026)		(0.0018)		(0.0018)	
N	100,215		100,215		100,215	
aic	22,286		5554		− 163,000	
bic	22,553		5820		− 163,000	

b. Births to White and Hispanic women	PTB		LBW		VLBW	
COVID-19, Race, Ethnicity						
COVID infect rate in Pregnancy trimester 01	0.0423	***	0.0335	***	0.0155	***
	(0.0019)		(0.0040)		(0.0025)	
Race/Ethnicity Hispanic (Any Race)	0.0091	***	0.0116	***	0.0034	***
	(0.0026)		(0.0034)		(0.0005)	
COVID infect rate trimester 01 x Hispanic	− 0.0018		0.0010		0.0023	***
	(0.0011)		(0.0007)		(0.0003)	
N	146,284		146,284		146,284	
aic	20,397		− 15,196		− 276,000	
bic	20,674		− 14,919		− 276,000	

A full table including all variables in available in Appendix 1

Includes all singleton births in Florida counties: Broward, Hillsborough, Orange, and Palm Beach. Jan-2018 to Dec-2022

*,**,***: t-test is significantly different from zero at the 0.05, 0.01, 0.001 level, respectively

Standard deviations in parentheses

Comparing White and Hispanic women, the latter had higher rates of PTB (0.91% points), LBW (1.16% points), and VLBW (0.34%, *p* < 0.001). There were, however, no significant interaction effects other than a 0.23% point increase in VLBW probability (*p* < 0.001).

## Discussion

Our study provides the first empirical evidence, to our knowledge, that community COVID-19 infection rates were associated with adverse pregnancy outcomes. Findings also offer insight into why multiple studies of pregnancy outcomes during the pandemic found adverse outcomes did not change, or even decreased (Grobman et al., 2023; Molina et al., 2022; Torche & Nobles, 2022). By considering the level of COVID-19 infections that pregnant women lived through during the first trimester, our study goes beyond those two-state tests to demonstrate that infection rates were associated with adverse outcome probabilities. Such findings are consistent with prior research linking social stressors to increases in PTB and LBW; particularly when events occur in the first trimester (Bond et al., 2019; Shapiro et al., 2013; Zhu et al., 2010). The scale of the impact is quite strong, with each 1% point increase in infections during the first trimester in the women’s county associated with a 5.16% point increase in PTB probabilities. This represents a 59% increase from the baseline PTB rate of 8.8% prior to the pandemic. This is in line with other studies of exposure to stressful events that show PTB increasing as much as 2.40 times (Zhu et al., 2010).

These findings demonstrate that community infection rates have an impact on pregnancy outcomes that cannot be solely attributed to the possibility of higher individual COVID-19 infections during community surges. Indeed, studies showing that first trimester individual infections resulted in the lowest odds of adverse outcomes (Doyle et al., 2022; Sevoyan et al., 2025; Son et al., 2021; Villar et al., 2021) would only dampen the significance of first trimester coefficients. If anything, this implies a potentially stronger impact of community infection levels.

Our study also reveals the significant impact that local COVID-19 infection rates have on pregnancy outcomes among Black women, underscoring a disproportionate effect compared to their White counterparts. Specifically, with each 1% point increase in infections during the first trimester, we observe increases in the probabilities of PTB, LBW, VLBW by 1.2, 1.6, and 1.3% points respectively for Black women. These increases correspond to a 9.6% rise from a baseline PTB rate of 12.51%, a 10% increase from a baseline LBW rate of 11.94%, and a substantial 23% increase from a baseline VLBW rate of 6.95%. As noted above, these disparities may be rooted in racial and ethnic differences in work settings. Although the woman’s occupation isn’t available in birth certificate data, we use their census tract to compute birth-weighted average tract employment profiles. In line with prior research (Asfaw, 2022; Faberman & Hartley, 2022), we find the average census tract share of workers employed in essential industries was higher for Black and Hispanic births. Further, the average census tract share of workers employed in positions least likely to be able to work from home was higher for Black births. This decreased ability to avoid work-based infection risk compounds already heighted concerns regarding infection among Black workers (Parker et al., 2022; Vargas et al., 2023); providing a possible explanation for disproportional impact.

## Limitations

Our DID analysis controlled for secular trends and temporal changes in the composition of study groups. This statistical approach is commonly used to evaluate outcomes before and after specific events, and has even been used in studies of birth outcomes during the pandemic (Caniglia et al., 2021). We enhance this approach by integrating local infection rates during the first trimester, rather than relying solely on binary pre-post pandemic indicators. The use of continuous measures of infection rates, as opposed to binary indicators, allows us to capture the varying intensity of COVID-19 over time. Binary treatment variables could fail to reflect this nuanced variation. Our choice of study model does present some limitations. Although our study doesn’t look at individual infection as other studies do (Khan et al., 2021; Prasannan et al., 2021; Torche & Nobles, 2022), our focus was on community-level stresses associated with the pandemic.

This study has several limitations. Set entirely in Florida, there may be state-level characteristics that need to be considered when generalizing results. For example, Florida has high proportions of Hispanic and immigrant women. Further, our study didn’t explore the full range on characteristics that could impact pregnancy outcomes, such as nativity and segregation. Although there is evidence that the pandemic era was associated with increases in individual stress levels in general, our study assumes that community infection rates were associated with the levels of stress experienced by individuals; This assumed relationship being the presumed path through which infection rates relate to stress levels, which in turn relate to pregnancy outcomes. Because COVID-19 infection data isn’t available below the county level, we could not test the significance of a more localized measure. However, most lockdown policies were implemented at the county level and a good deal of stress may be related to where women work and not where they live. Although maternal occupation data is not available, it would have helped to test whether occupation and ability to telecommute moderate pandemic stresses. Although this study didn’t test the interaction between the timing of infection surges and lockdowns, Florida was one of the earliest states to lift restrictions.

## Conclusion

We leveraged a sophisticated database coupled with rigorous statistical techniques to explore the impact of COVID-19 infection rates on pregnancy outcomes and disparities. We found conclusive evidence that county infection rates adversely impacted pregnancies, with each 1% point increase in COVID-19 cases during the first trimester associated with percentage point increases of 5.16 PTB, 4.35 LBW and 2.59 VLBW. We also found conclusive evidence of widening disparities, with Black women experiencing a further increase of 1.21% points in PTB, 1.57 for LBW, and 1.28 for VLBW for each 1% point increase in first trimester infections. Our findings are relevant to future pandemic policy decisions, showing that infection surges are associated with increases in adverse pregnancy outcomes and broadening disparities.

## Supplementary Information

Below is the link to the electronic supplementary material.

Supplementary Material 1

## Data Availability

Source data is publicly available on-line for free.
