# Supplementary material for: Impact of COVID-19 Infection Rates on Pregnancy Outcomes and Disparities in Florida
Source: Matern Child Health J. 2025 Oct 28;29(12):1736–47. doi: 10.1007/s10995-025-04184-6 (PMC12675694; doi:10.1007/s10995-025-04184-6)
Supplement: Supplementary file 1 — Supplementary Material 1 [file 10995_2025_4184_MOESM1_ESM.docx]

Title: Impact of COVID-19 infection rates on pregnancy outcomes and disparities in Florida.

Appendix 1. Parallel Trend Test

The parallel trends assumption is a fundamental prerequisite for the validity of difference-in-differences (DiD) analysis. This assumption posits that, in the absence of the treatment, the outcome trends for the treatment and control groups would have been the same. To validate this assumption for our study, we conducted a parallel trends test. This test examines whether the pre-treatment outcome trends are statistically indistinguishable across the groups defined by racial/ethnic categories. For our study, we designate March 1, 2020, as the start date of the COVID-19 pandemic in Florida, aligning with the CDC's recognition of the pandemic's presence in the state. This is crucial for accurately defining our pre-treatment period. We exclude the post-pandemic period from visual presentations due to the varying intensity of COVID-19 exposure over time. Since a simple treatment time dummy variable might not adequately capture these monthly variations in exposure, representing the post-period could potentially lead to misleading interpretations.

Accordingly, our pre-period trend analysis includes graphical representations (Figure 1 through Figure 6), and an F-test to determine the statistical significance of the pre-period coefficients (Table 1). As depicted in the graphs below, the F-test does not reveal any significant deviations in trends among the groups prior to March 2020, supporting the plausibility of the parallel trend assumption in our study context.

.

Figure 1. Parallel Trend Test: White-Black, PTB


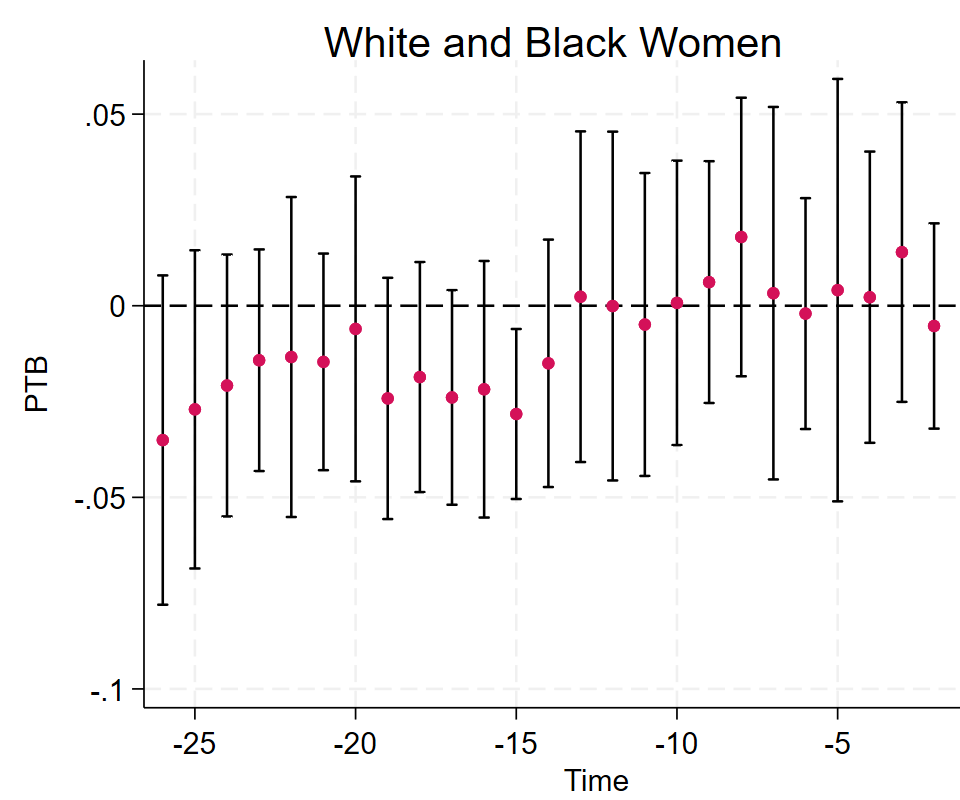


Figure 2. Parallel Trend Test: White-Black, LBW


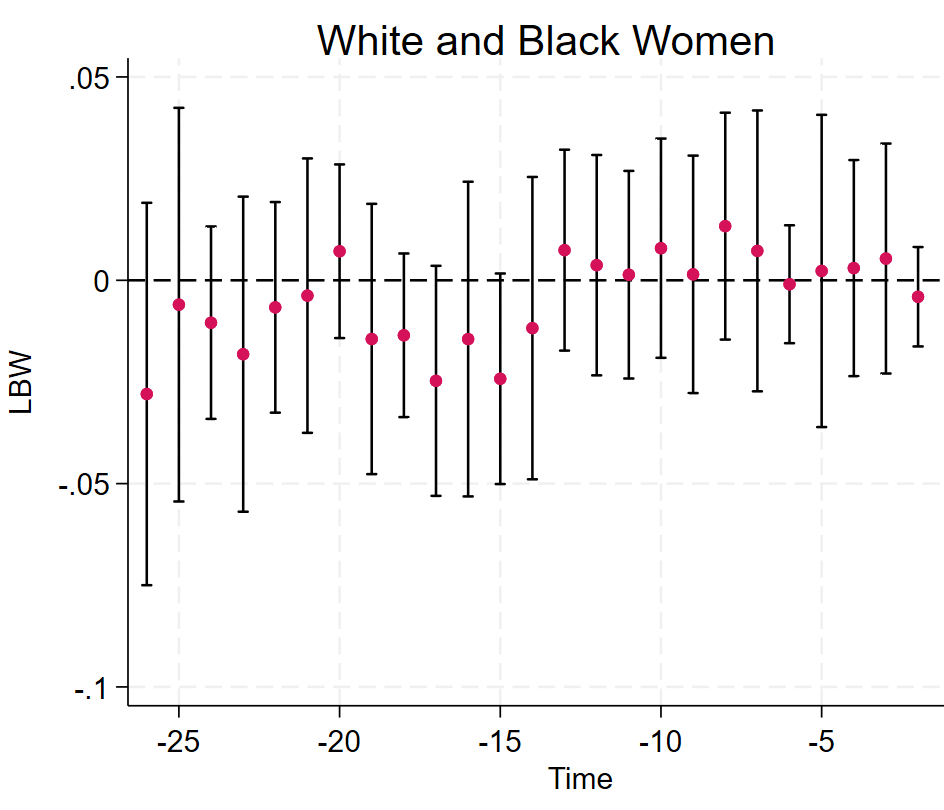
.

Figure 3. Parallel Trend Test: White-Black, VLBW


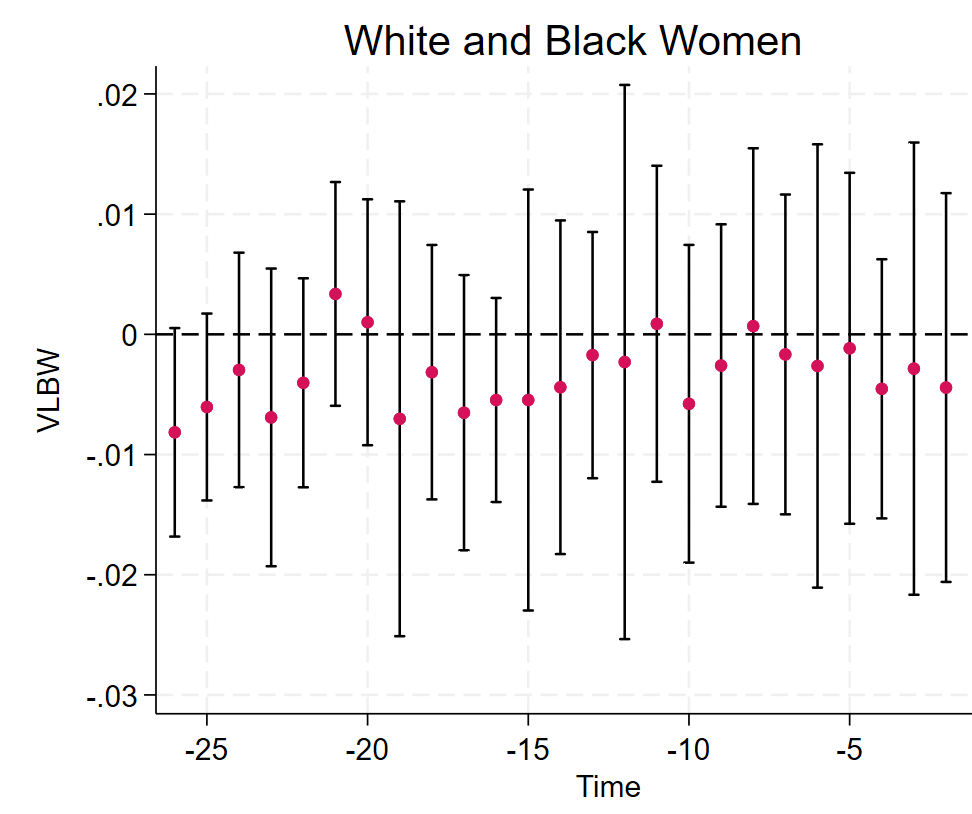


Figure 4. Parallel Trend Test: White-Hispanic, PTB


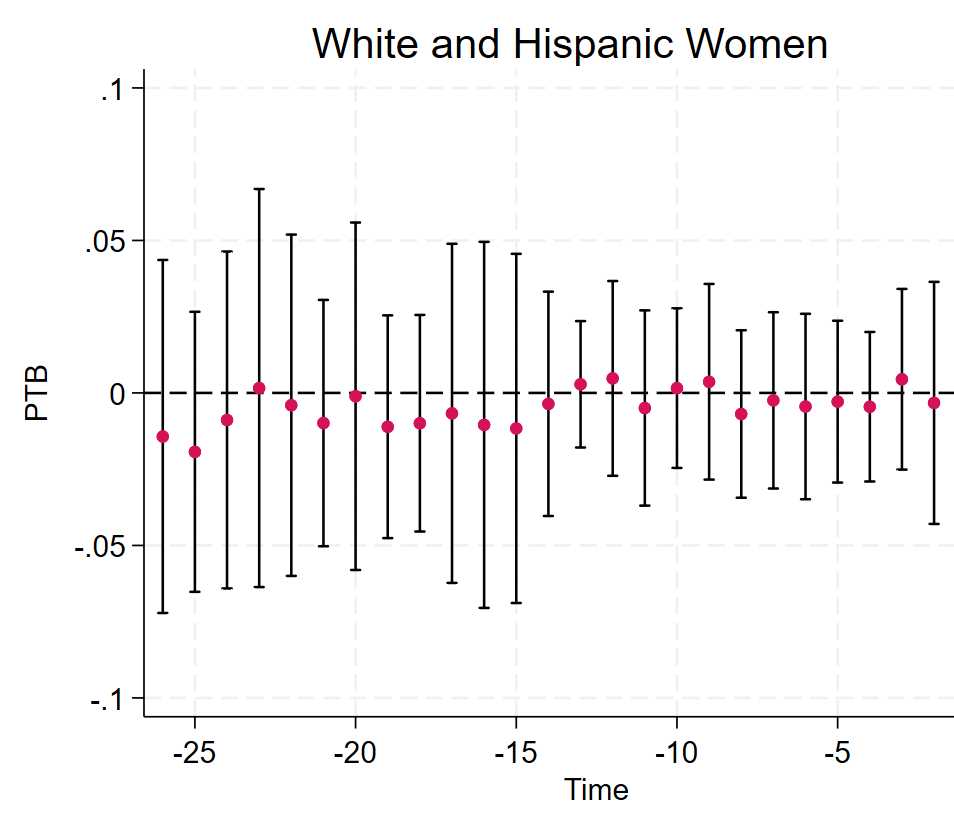


Figure 5. Parallel Trend Test: White-Hispanic, LBW


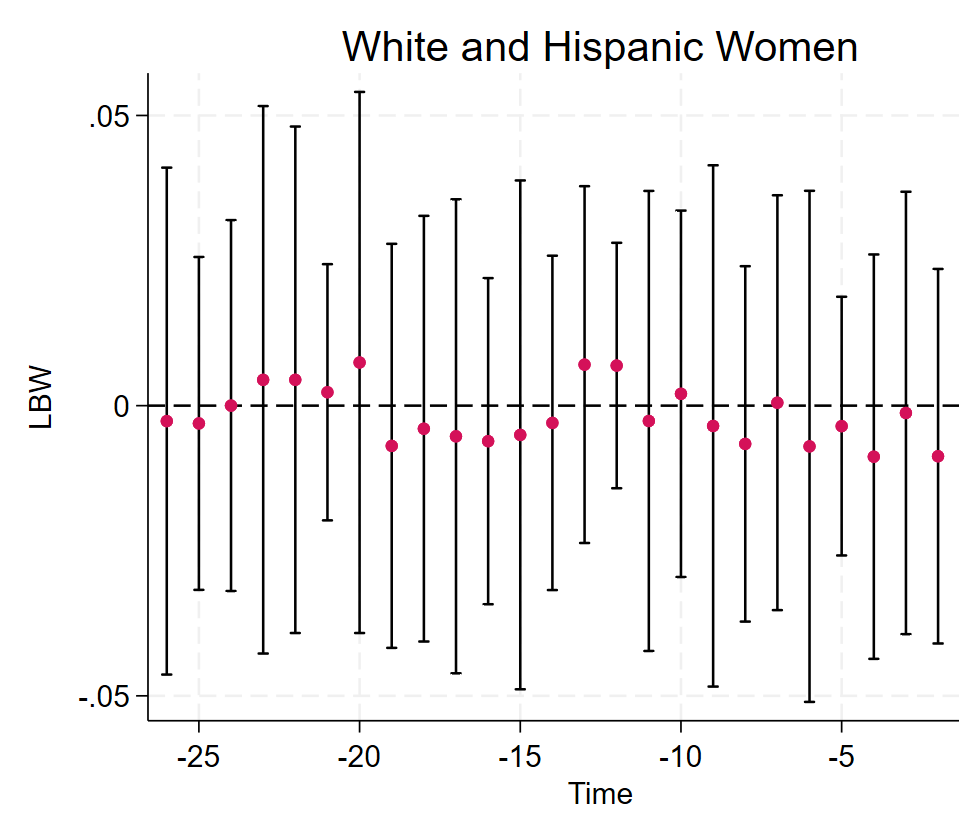


Figure 6. Parallel Trend Test: White-Hispanic, VLBW


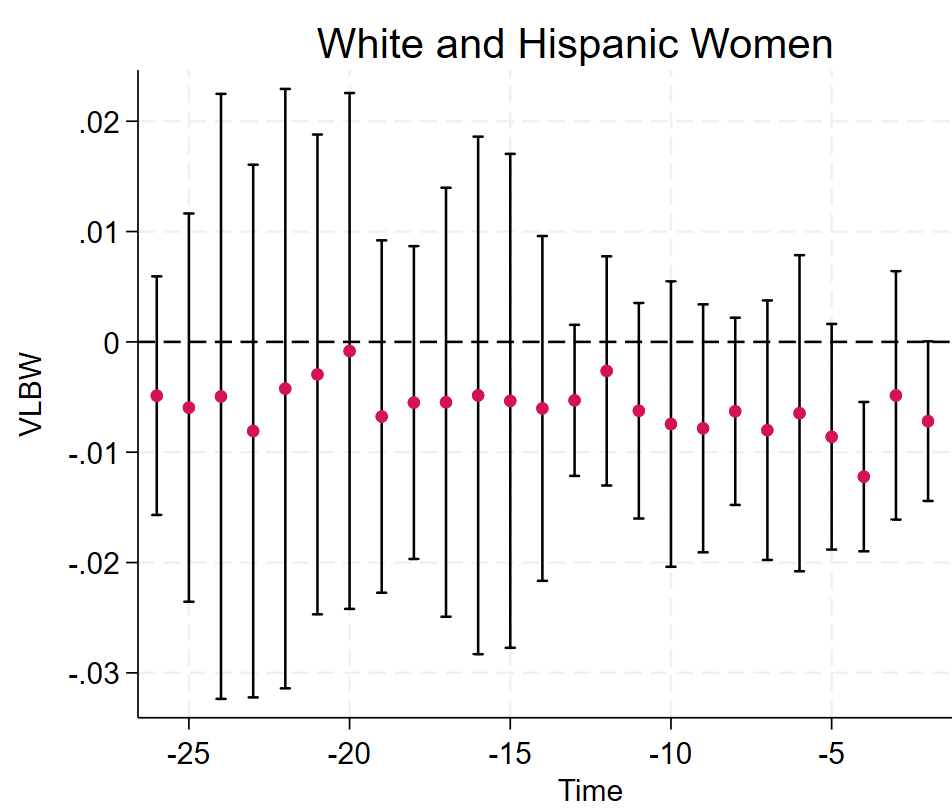


Table 1. F-Test Results for Parallel Trends Assumption Before COVID-19 Pandemic

| **Group Comparison** | **PTB (Prob > F)** | **LBV (Prob > F)** | **VLBV (Prob > F)** |
| --- | --- | --- | --- |
| White and Black Women | 0.1895 | 0.5688 | 0.3815 |
| White and Hispanic Women | 0.2482 | 0.8676 | 0.4474 |

.

Appendix 2. Full Regression Tables

Table 2. Full version of Table 3b. Births to White and Black mothers.

|  | PTB |  | LBW |  | VLBW |  |
| --- | --- | --- | --- | --- | --- | --- |
| **COVID-19, Race, Ethnicity** |  |  |  |  |  |  |
| COVID infect rate in Pregnancy trimester 01 | 0.0525 | *** | 0.0414 | *** | 0.0235 | *** |
|  | (0.0043) |  | (0.0044) |  | (0.0045) |  |
| Mother Race/Ethnicity Black Non-Hispanic | 0.0314 | *** | 0.0448 | *** | 0.0103 | *** |
|  | (0.0028) |  | (0.0029) |  | (0.0006) |  |
| COVID infect rate trimester 01 x Black | 0.0121 | *** | 0.0157 | *** | 0.0128 | *** |
|  | (0.0026) |  | (0.0018) |  | (0.0018) |  |
| **Maternal** |  |  |  |  |  |  |
| Age 00-19 | -0.0402 | *** | -0.0226 |  | -0.0037 |  |
|  | (0.0049) |  | (0.0132) |  | (0.0025) |  |
| Age 20-24 | -0.0311 | *** | -0.0203 | *** | -0.0054 | *** |
|  | (0.0036) |  | (0.0035) |  | (0.0012) |  |
| Age 25-29 | -0.0110 | *** | -0.0080 | *** | -0.0022 | *** |
|  | (0.0009) |  | (0.0012) |  | (0.0005) |  |
| Age 35-39 | 0.0134 | *** | 0.0074 | ** | 0.0024 | *** |
|  | (0.0030) |  | (0.0028) |  | (0.0007) |  |
| Age 40-up | 0.0395 | *** | 0.0316 | *** | 0.0068 | ** |
|  | (0.0035) |  | (0.0036) |  | (0.0021) |  |
| Education Attainment sub-HS | 0.0392 | *** | 0.0370 | *** | 0.0021 |  |
|  | (0.0059) |  | (0.0064) |  | (0.0015) |  |
| Education Attainment HS | 0.0183 | *** | 0.0141 | *** | 0.0028 | *** |
|  | (0.0035) |  | (0.0042) |  | (0.0008) |  |
| Pre-Pregnancy BMI Underweight | 0.0208 | *** | 0.0341 | *** | 0.0050 | *** |
|  | (0.0045) |  | (0.0023) |  | (0.0003) |  |
| Pre-Pregnancy BMI Overweight | 0.0049 | * | -0.0055 | *** | 0.0014 | * |
|  | (0.0020) |  | (0.0014) |  | (0.0006) |  |
| Pre-Pregnancy BMI Obese | 0.0222 | *** | -0.0022 |  | 0.0034 | *** |
|  | (0.0029) |  | (0.0013) |  | (0.0006) |  |
| **Insurance and Family** |  |  |  |  |  |  |
| Payor - Medicaid | -0.0002 |  | 0.0092 | ** | -0.0029 |  |
|  | (0.0055) |  | (0.0032) |  | (0.0039) |  |
| Payor - Private | -0.0076 |  | 0.0025 |  | -0.0044 |  |
|  | (0.0074) |  | (0.0040) |  | (0.0046) |  |
| Payor - Self Pay | -0.0297 | ** | -0.0125 |  | -0.0083 |  |
|  | (0.0112) |  | (0.0080) |  | (0.0043) |  |
| Married | -0.0063 | * | -0.0122 | *** | -0.0016 |  |
|  | (0.0029) |  | (0.0028) |  | (0.0013) |  |
| Father Name Missing | 0.0242 | *** | 0.0183 | *** | 0.0045 | *** |
|  | (0.0014) |  | (0.0031) |  | (0.0012) |  |
| **Community** |  |  |  |  |  |  |
| Popn % Not Hispanic (White race only) | -0.00001 |  | 0.00001 |  | -0.00002 |  |
|  | (0.00007) |  | (0.00008) |  | (0.00002) |  |
| Popn % Not Hispanic (Black race only) | 0.00024 | * | 0.00021 | ** | 0.00004 |  |
|  | (0.00010) |  | (0.00008) |  | (0.00005) |  |
| Popn % Not White nor Black | 0.00005 |  | 0.00003 |  | -0.00004 |  |
|  | (0.00018) |  | (0.00004) |  | (0.00007) |  |
| % Unemployment | 0.0008 | * | 0.0010 | *** | 0.0001 |  |
|  | (0.0004) |  | (0.0003) |  | (0.0001) |  |
| Median household income (ln) | -0.0032 | * | -0.0048 | *** | -0.0027 | *** |
|  | (0.0014) |  | (0.0014) |  | (0.0005) |  |
| **Controls** |  |  |  |  |  |  |
| Baby DOB Year=2019 | 0.0011 |  | -0.0023 | ** | -0.0007 | * |
|  | (0.0010) |  | (0.0008) |  | (0.0004) |  |
| Baby DOB Year=2020 | -0.0013 |  | -0.0021 | * | -0.0002 |  |
|  | (0.0011) |  | (0.0009) |  | (0.0009) |  |
| Baby DOB Year=2021 | -0.0412 | *** | -0.0356 | *** | -0.0223 | *** |
|  | (0.0084) |  | (0.0066) |  | (0.0053) |  |
| Baby DOB Year=2022 | -0.0611 | *** | -0.0498 | *** | -0.0328 | *** |
|  | (0.0070) |  | (0.0041) |  | (0.0053) |  |
| Constant | 0.0911 | *** | 0.0946 | *** | 0.0409 | *** |
|  | (0.0201) |  | (0.0108) |  | (0.0089) |  |
| N | 100,215 |  | 100,215 |  | 100,215 |  |
| aic | 22,286 |  | 5,554 |  | -163,000 |  |
| bic | 22,553 |  | 5,820 |  | -163,000 |  |

Notes.

Time periods: "Pre" includes Jan-2018 to Feb-2020; "Post" includes Mar-2020 to Dec-2022. Includes all singleton births in Florida counties: Broward, Hillsborough, Orange, and Palm Beach. Jan-2018 to Dec-2022.

Base levels: Baby DOB (date of birth) base year is 2018. Maternal age base is 30-34. Pre-Pregnancy BMI base level is "Normal". Education attainment base level is "over high school" (associates, bachelors, etc)

*,**,***: t-test is significantly different from zero at the .05, .01, .001 level, respectively.

Standard deviations in parentheses.

Table 2. Full version of Table 3b. Births to White and Hispanic mothers.

|  | PTB |  | LBW |  | VLBW |  |
| --- | --- | --- | --- | --- | --- | --- |
| **COVID-19, Race, Ethnicity** |  |  |  |  |  |  |
| COVID infect rate in Pregnancy trimester 01 | 0.0423 | *** | 0.0335 | *** | 0.0155 | *** |
|  | (0.0019) |  | (0.0040) |  | (0.0025) |  |
| Mother Race/Ethnicity Hispanic (Any Race) | 0.0091 | *** | 0.0116 | *** | 0.0034 | *** |
|  | (0.0026) |  | (0.0034) |  | (0.0005) |  |
| COVID infect rate trimester 01 x Hispanic | -0.0018 |  | 0.0010 |  | 0.0023 | *** |
|  | (0.0011) |  | (0.0007) |  | (0.0003) |  |
| **Maternal** |  |  |  |  |  |  |
| Age 00-19 | 0.0010 |  | 0.0062 | ** | 0.0006 |  |
|  | (0.0023) |  | (0.0023) |  | (0.0028) |  |
| Age 20-24 | -0.0098 | *** | -0.0009 |  | -0.0022 | *** |
|  | (0.0027) |  | (0.0017) |  | (0.0006) |  |
| Age 25-29 | -0.0066 | *** | -0.0029 |  | -0.0009 |  |
|  | (0.0005) |  | (0.0015) |  | (0.0005) |  |
| Age 35-39 | 0.0139 | *** | 0.0091 | *** | 0.0021 | *** |
|  | (0.0025) |  | (0.0009) |  | (0.0003) |  |
| Age 40-up | 0.0412 | *** | 0.0319 | *** | 0.0057 | *** |
|  | (0.0031) |  | (0.0019) |  | (0.0015) |  |
| Education Attainment sub-HS | 0.0092 |  | 0.0055 |  | -0.0006 |  |
|  | (0.0047) |  | (0.0066) |  | (0.0015) |  |
| Education Attainment HS | 0.0084 | ** | 0.0036 |  | 0.0006 |  |
|  | (0.0026) |  | (0.0029) |  | (0.0005) |  |
| Pre-Pregnancy BMI Underweight | 0.0153 | * | 0.0283 | *** | 0.0042 | *** |
|  | (0.0060) |  | (0.0063) |  | (0.0007) |  |
| Pre-Pregnancy BMI Overweight | 0.0034 | * | -0.0051 | *** | 0.0010 | *** |
|  | (0.0015) |  | (0.0004) |  | (0.0002) |  |
| Pre-Pregnancy BMI Obese | 0.0244 | *** | 0.0022 |  | 0.0040 | *** |
|  | (0.0038) |  | (0.0017) |  | (0.0003) |  |
| **Insurance and Family** |  |  |  |  |  |  |
| Payor - Medicaid | 0.0049 |  | 0.0017 |  | 0.0017 |  |
|  | (0.0038) |  | (0.0012) |  | (0.0017) |  |
| Payor - Private | -0.0036 |  | -0.0049 | *** | -0.0014 |  |
|  | (0.0020) |  | (0.0014) |  | (0.0016) |  |
| Payor - Self Pay | -0.0212 | *** | -0.0201 | *** | -0.0056 | *** |
|  | (0.0028) |  | (0.0026) |  | (0.0016) |  |
| Married | -0.0063 | ** | -0.0092 | *** | -0.0010 |  |
|  | (0.0022) |  | (0.0016) |  | (0.0010) |  |
| Father Name Missing | 0.0310 | *** | 0.0232 | *** | 0.0053 | *** |
|  | (0.0022) |  | (0.0024) |  | (0.0007) |  |
| **Community** |  |  |  |  |  |  |
| Popn % Not Hispanic (White race only) | -0.00009 |  | -0.00002 |  | -0.00001 |  |
|  | (0.00005) |  | (0.00003) |  | (0.00002) |  |
| Popn % Not Hispanic (Black race only) | 0.00021 | * | 0.00030 | *** | 0.00007 | * |
|  | (0.00009) |  | (0.00003) |  | (0.00003) |  |
| Popn % Not White nor Black | -0.00007 |  | 0.00003 |  | -0.00007 | *** |
|  | (0.00020) |  | (0.00005) |  | (0.00002) |  |
| % Unemployment | -0.0003 |  | 0.0000 |  | 0.0000 |  |
|  | (0.0002) |  | (0.0001) |  | (0.0000) |  |
| Median household income (ln) | -0.0035 |  | -0.0026 |  | -0.0009 | ** |
|  | (0.0021) |  | (0.0031) |  | (0.0003) |  |
| **Controls** |  |  |  |  |  |  |
| Baby DOB Year=2019 | 0.0038 | *** | -0.0010 |  | -0.0003 |  |
|  | (0.0011) |  | (0.0015) |  | (0.0012) |  |
| Baby DOB Year=2020 | -0.0034 | *** | -0.0050 | * | -0.0028 | *** |
|  | (0.0005) |  | (0.0021) |  | (0.0006) |  |
| Baby DOB Year=2021 | -0.0297 | *** | -0.0276 | *** | -0.0155 | *** |
|  | (0.0054) |  | (0.0049) |  | (0.0026) |  |
| Baby DOB Year=2022 | -0.0479 | *** | -0.0407 | *** | -0.0213 | *** |
|  | (0.0034) |  | (0.0029) |  | (0.0021) |  |
| Constant | 0.0993 | *** | 0.0790 | * | 0.0174 | *** |
|  | (0.0248) |  | (0.0344) |  | (0.0041) |  |
| N | 146,284 |  | 146,284 |  | 146,284 |  |
| aic | 20,397 |  | -15,196 |  | -276,000 |  |
| bic | 20,674 |  | -14,919 |  | -276,000 |  |

Notes.

Time periods: "Pre" includes Jan-2018 to Feb-2020; "Post" includes Mar-2020 to Dec-2022. Includes all singleton births in Florida counties: Broward, Hillsborough, Orange, and Palm Beach. Jan-2018 to Dec-2022.

Base levels: Baby DOB (date of birth) base year is 2018. Maternal age base is 30-34. Pre-Pregnancy BMI base level is "Normal". Education attainment base level is "over high school" (associates, bachelors, etc)

*,**,***: t-test is significantly different from zero at the .05, .01, .001 level, respectively.

Standard deviations in parentheses.
